# Progressive vestibular dysfunction in unilateral acute otitis media with serous labyrinthitis: a case report

**DOI:** 10.1186/s13256-025-05651-y

**Published:** 2025-11-27

**Authors:** Liqing Chen, Yunwen Wu, Yan Li, Xiaoyan Zhang, Jinwei Du, Nannan Zhang, Zhiyuan Tang, Qiang WU, Qing Yuan

**Affiliations:** 1 Department of Otolaryngology, Head and Neck Surgery, Shenzhen University General Hospital, Shenzhen, 518055 China; 2 Shenzhen, Guangdong province, China

**Keywords:** Vertigo, Labyrinthitis, Vestibular function

## Abstract

**Objective:**

This study aimed to investigate vestibular function changes in patients with serous labyrinthitis and to discuss the possible mechanisms involved.

**Patient presentation:**

A 34-year-old female patient of Chinese ethnicity presented with progressive vestibular dysfunction and unilateral acute otitis media complicated by serous labyrinthitis. Upon diagnosis, systemic antibiotic and glucocorticoid therapy was initiated; ceftriaxone sodium (2 g) and dexamethasone (10 mg) were administered via intravenous infusion once daily for 5 days, complemented by hydroxymethazoline and mometasone furoate nasal sprays as adjunctive antiinflammatory measures. Within a week, audiometric thresholds returned to normal, nystagmus intensity progressively diminished, and vertigo resolved entirely.

**Conclusion:**

Patients with vertigo and labyrinthitis secondary to acute otitis media typically exhibit variable vestibular symptoms, with unidirectional irritative nystagmus serving as a cardinal diagnostic sign. Integration of audiological and vestibular function assessments, including meticulous surveillance of clinical evolution, is paramount to prevent misdiagnosis or treatment delay. The differential impacts of toxic versus inflammatory mediators on inner ear physiology may render early combination therapy with antibiotics and glucocorticoids particularly efficacious.

## Introduction

Acute otitis media (AOM) is defined by the abrupt onset and rapid progression of inflammatory manifestations in the middle ear. The most common aetiologies of AOM include infections with *Streptococcus pneumoniae*, *Haemophilus influenzae*, or *Moraxella catarrhalis* [1]. The early initiation of antibiotic therapy in the management of AOM has resulted in a significant reduction in the incidence of labyrinthitis [2]. Serous labyrinthitis represents one of the most frequent complications of AOM.

Recently, cases of serous labyrinthitis have been infrequently reported in the otolaryngological literature [3, 4]. However, the pathophysiological mechanisms governing progressive vestibular dysfunction in these patients remain incompletely characterized. In this study, we document unique findings from videonystagmography (VNG) and video head impulse testing (vHIT) in a patient with serous labyrinthitis and provide an analysis of potential mechanistic pathways.

## Case presentation

### Medical history

A 34-year-old female patient presented to our clinic with progressive right-sided vertigo and hearing loss; 2 days after an upper respiratory tract infection, the patient developed right ear fullness, low-frequency tinnitus, and vertigo following vigorous nose blowing. At presentation, she denied fever, irritability, otalgia, headache, otorrhea, nausea, or vomiting. There was no history of ear disease, ear surgery, recent head trauma, or chronic systemic disorders.

### Physical examination

Nasal examination revealed nasal mucosa congestion, a deviated nasal septum to the left, and purulent discharge in the middle and inferior nasal meati. The external auditory canals were patent bilaterally, with intact tympanic membranes. Otoscopic evaluation revealed right tympanic membrane bulging and hyperemia, and the left ear appeared normal. Spontaneous nystagmus was noted as slight right-beating horizontal nystagmus. Ocular motor tests revealed normal saccadic eye movements and smooth pursuit. Rombergs sign was negative, and the horizontal head thrust test and head shake test results were both negative.

### Accessory examination

Multiple studies, including audiograms, tympanograms, electronystagmography, and vHIT, were performed along with imaging to aid in the diagnosis.

### Audiograms

**Fig. 1:**
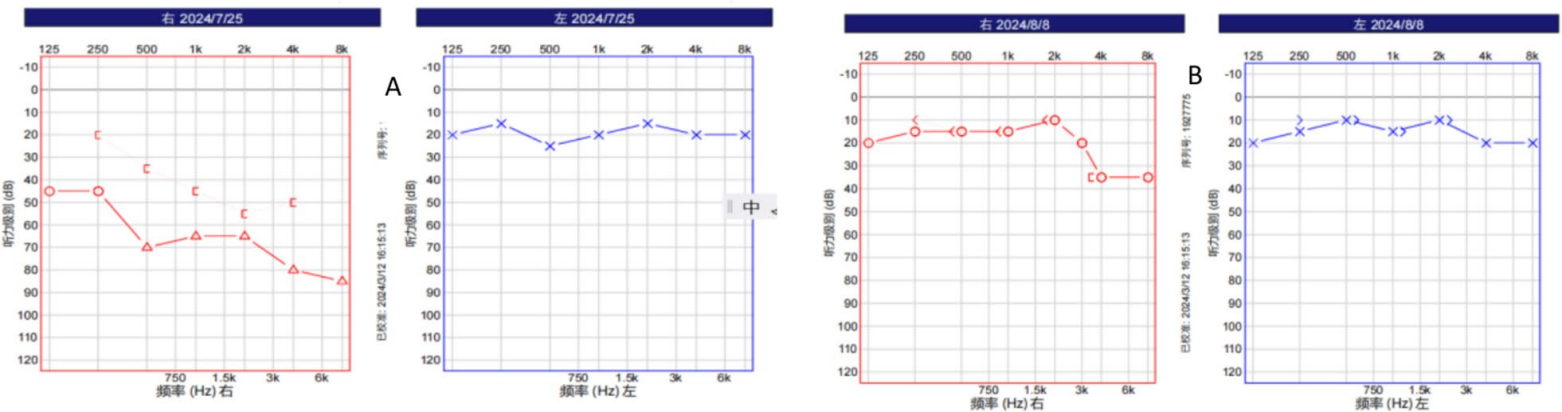
Serial pure-tone audiometry results showing a therapeutic response in the right ear with mixed hearing loss. **A** Initial evaluation (25 July 2024) revealed right ear mixed hearing loss (air–bone gap: 24 dB) with normal left ear thresholds. **B** Posttreatment assessment (8 August 2024) revealed complete normalization of the right ear thresholds

Pure-tone audiometry (PTA) revealed air and bone conduction thresholds consistent with mixed hearing loss in the right ear (air conduction: 70 dB HL; bone conduction: 46 dB HL; air–bone gap: 24 dB HL), whereas the left ear presented normal hearing (20 dB HL). (Fig. 1.)

### Tympanograms

**Fig. 2:**
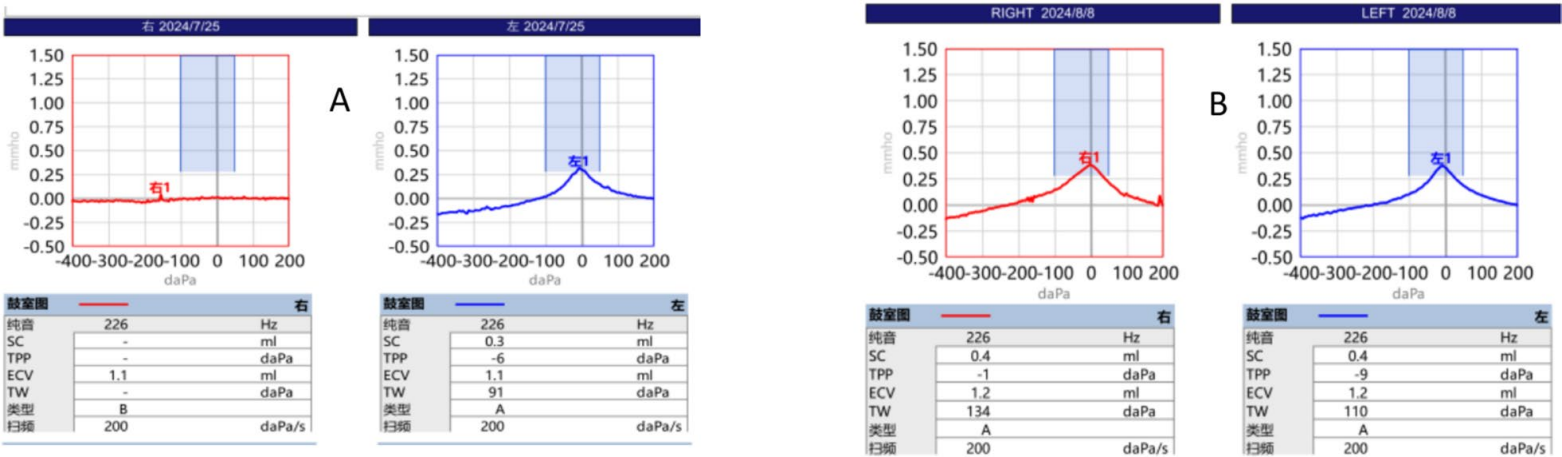
Serial tympanometry results showing the resolution of middle ear dysfunction. **A** The initial test (25 July 2024) revealed a type B tympanogram (flat curve) in the right ear, indicating middle ear effusion, with a normal type A tympanogram in the left ear. **B** Posttreatment test (8 August 2024) revealed bilateral normal type A tympanograms, demonstrating complete resolution

The right ear was type B; the left ear was type A (Fig. 2).

### Videonystagmography (VNG)

**Fig. 3:**
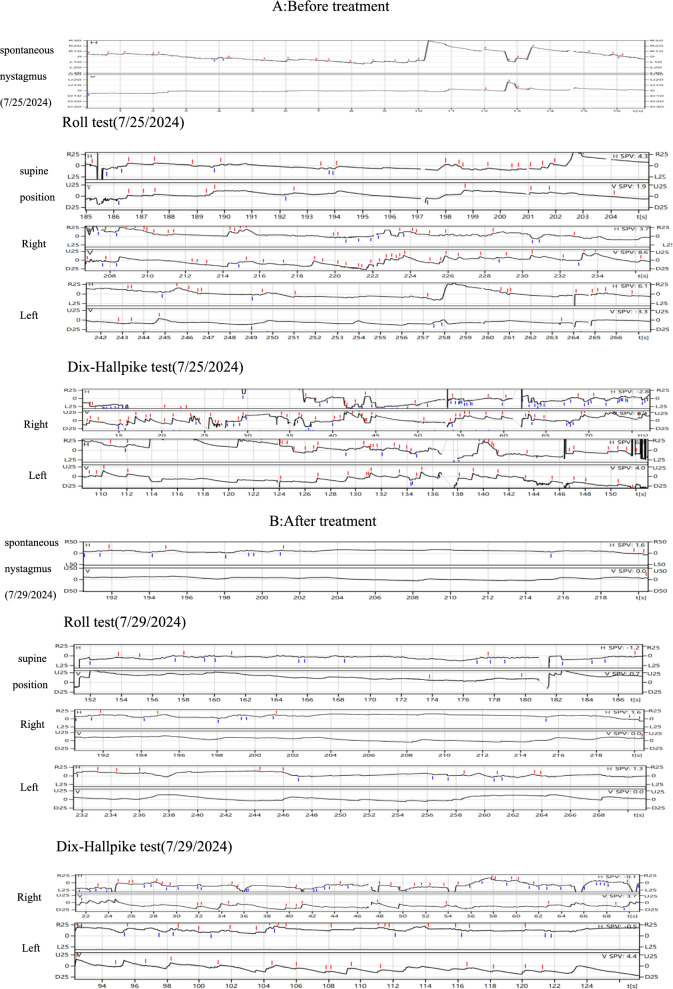
Videonystagmography findings before and after treatment. **A** Initial assessment (25 July 2024) revealed spontaneous right-beating horizontal nystagmus (3–4° per second), type II smooth pursuit impairment, decreased right optokinetic response, persistent upbeat (7–8° per second) and right-beating horizontal nystagmus (4–6° per second) on positional testing, and intolerance. **B** Posttreatment evaluation demonstrated resolution of spontaneous nystagmus, normalized optokinetic responses, and mild residual positional nystagmus (3–4° per second)

The patient’s eye movements were examined at various head positions and recorded using goggles installed with an infrared camera (VertiGoggles (ZT-VNG-II), Shanghai ZEHNIT Medical Technology Co., Ltd., Shanghai, China). Spontaneous nystagmus was horizontal to the right 3–4° per second, and saccades and gaze were normal; smooth pursuit was type II. In the optokinetic test, the right side was decreased and the left side was normal; in the Dix–Hallpike test: persistent upbeat 7–8° per second on the right side, persistent horizontal nystagmus to the right 4–5° per second, and upbeat 7–8° per second on the left side; and in the roll test, persistent horizontal nystagmus to the right 5–6° per second in the supine position, persistent horizontal nystagmus to the right 4–5° per second on the left side, and persistent up beat 7–8° per second on the right side. The patient was intolerant and unable to cooperate with the caloric test prior to treatment. After treatment, the degree of nystagmus gradually decreased, and the direction of nystagmus changed (Fig. 3).

### vHIT

**Fig. 4:**
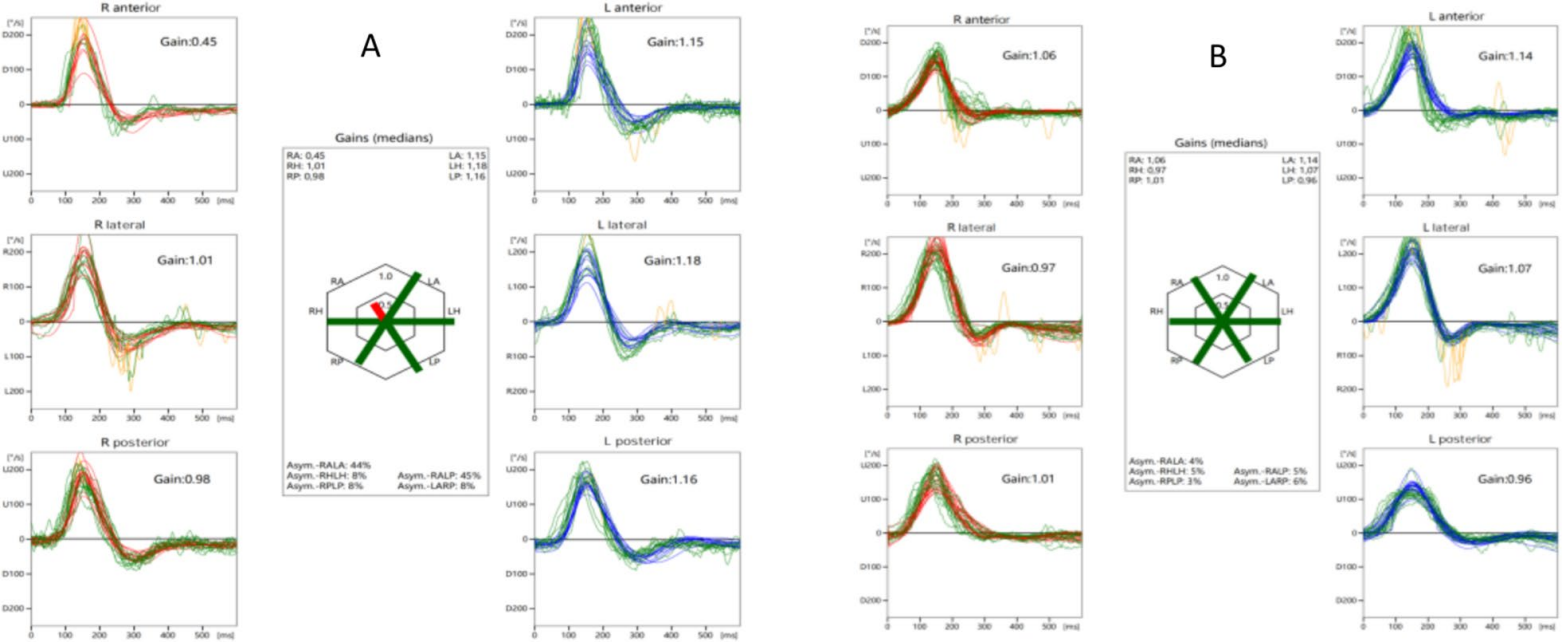
Video head impulse test (vHIT) results showing vestibular function recovery. **A** Initial testing (25 July 2024) demonstrated reduced vestibulo–ocular reflex (VOR) gain (0.45) in the affected posterior semicircular canal without corrective saccades. **B** Posttreatment evaluation (29 July 2024) revealed a normalized VOR gain (0.85) in all canals

vHIT was performed by a trained technician via an Otometrics Impulse (VertiGoggles (ZT-VNG-II), Shanghai ZEHNIT Medical Technology Co., Ltd., Shanghai, China) to record the vestibular–ocular reflex (VOR). During vHIT, the patient visualized an eye-level target on the wall at a distance of 1–1.5 m and wore tight-fitting goggles. The technicians stood behind the patient with their hands placed on the vertex and chin of the patient. The head impulses were rapid, small, and unpredictable. The corrective saccades were categorized according to their appearance as covert or overt. The initial vHIT assessment revealed a diminished vestibulo–ocular reflex (VOR) gain of 0.45 in the anterior semicircular canal on the affected side. The gain asymmetry between the right anterior semicircular canal and the left posterior semicircular canal was 45%, without overt or covert saccades, and returned to normal following treatment. In the present study, the pathological value for VOR gain was defined as a horizontal vHIT gain < 0.8 or vertical vHIT gain < 0.7, in accordance with Isaac *et al*.’s suggestion for normal horizontal VOR gain values [5]. The patterns of the video head impulse test abnormalities are shown in Fig. 4.

### The 64-slice Siemens computed tomography (CT) images of the middle ear

**Fig. 5:**
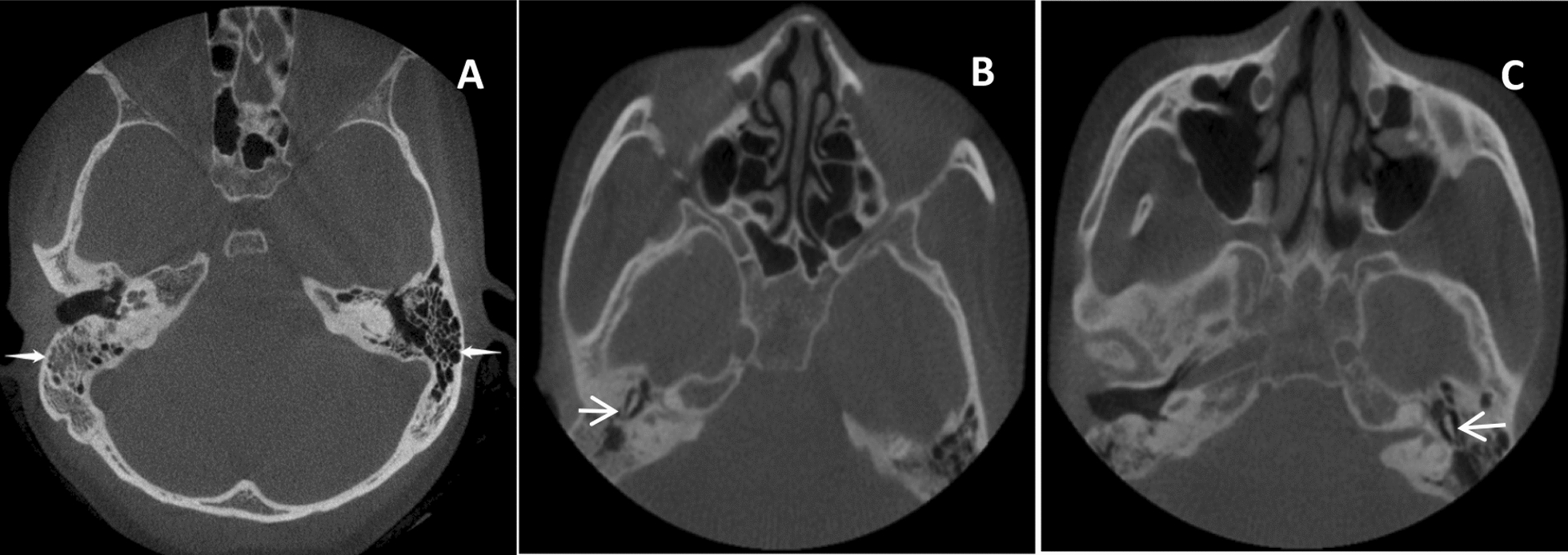
Temporal bone computed tomography findings. **A** Axial section demonstrating opacification of right mastoid air cells (arrow), with intact contralateral pneumatization. **B**, **C** Coronal sections showing unobstructed bilateral mesotympanums (arrows), without evidence of middle ear effusion or ossicular pathologies

The 64-slice Siemens CT images demonstrated increased density shadows in the right mastoid air cell and mesotympanum; the contralateral mastoid was pneumatized well (Fig. 5).

### Siemens 3.0 Tesla magnetic resonance imaging (MRI) of the middle ear

**Fig. 6:**
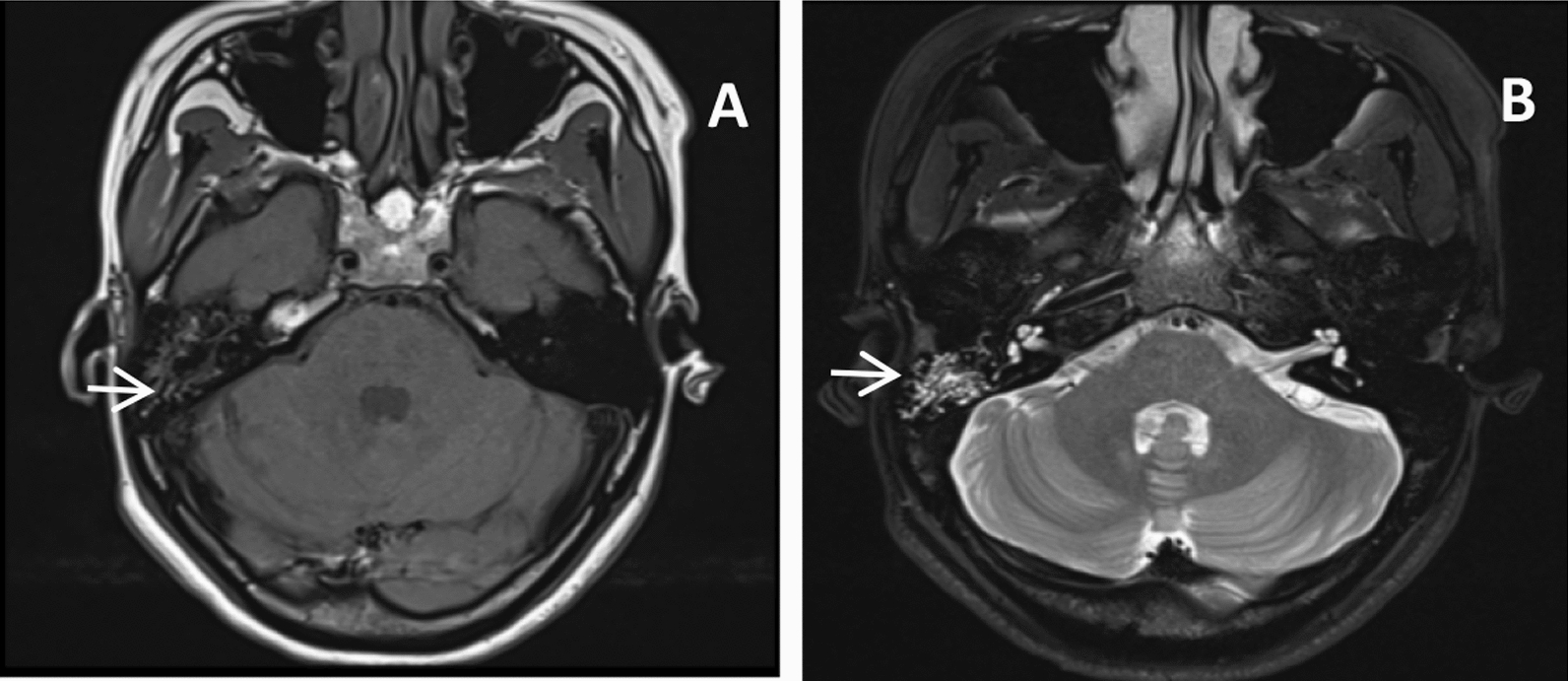
Pretreatment magnetic resonance imaging findings of right mastoid pathology. **A** T1-weighted imaging reveals a hypointense signal (arrow) within right mastoid air cells. **B** Corresponding T2-weighted image shows a hyperintense signal (arrow), indicative of fluid content. Inner ear structures, the cerebellopontine angle, and the brainstem maintain normal signal intensity

The Siemens 3.0 Tesla MRI demonstrated patchy, homogeneous signals in the right mastoid air chamber. T1WI revealed a low signal, T2WI revealed a high signal, and no abnormal signals were observed in the inner ear, cerebellopontine angle, or brainstem (Fig. 6).

### Endoscopy

**Fig. 7:**
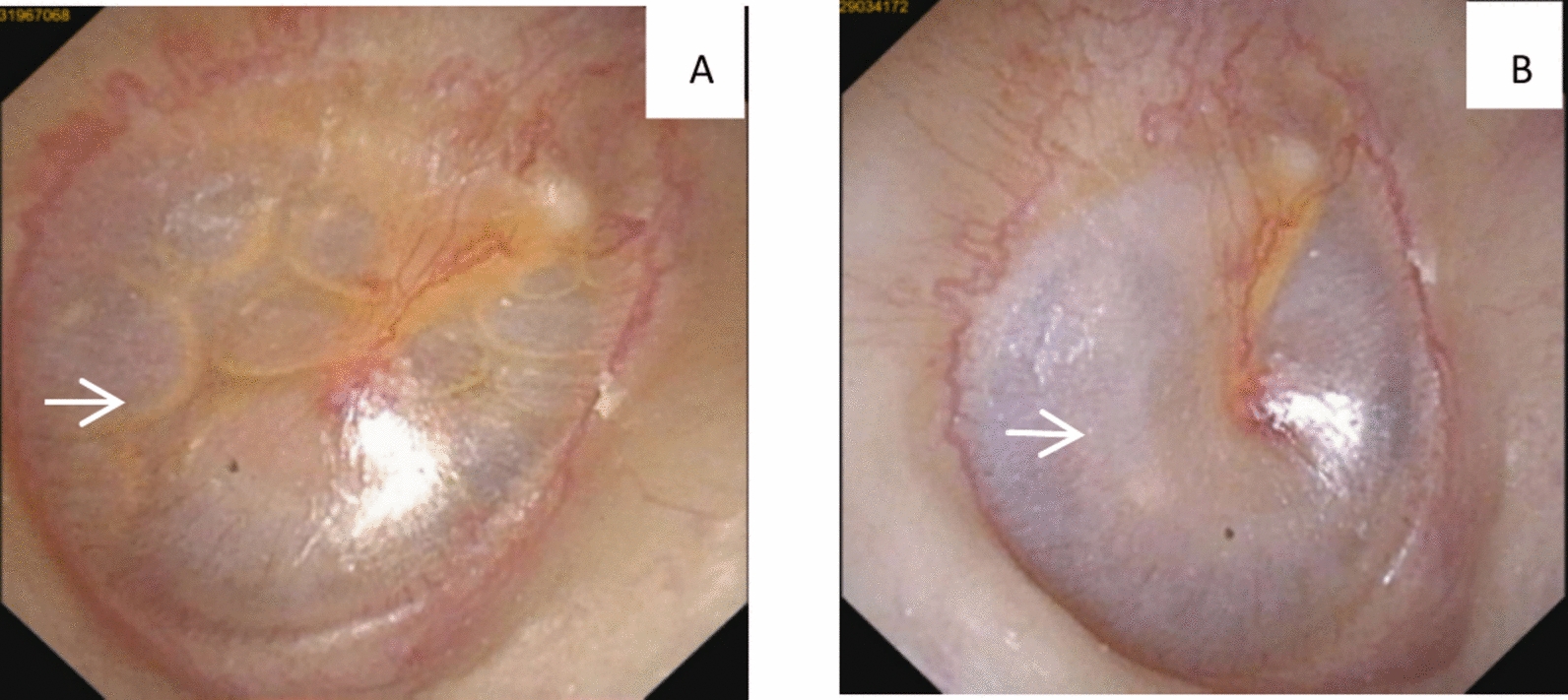
Endoscopic assessment of tympanic membrane morphology before and after treatment. **A** Initial endoscopic examination (25 July 2024) revealed an intact right tympanic membrane with middle ear fluid (arrow). **B** Follow-up examination (28 July 2024) showing complete resolution of fluid and normalization of tympanic membrane morphology (arrow)

Endoscopy revealed that the right tympanic membrane was complete, and liquid accumulation was observed in the right middle tympanic region. The liquid accumulation in the right middle tympanic membrane disappeared after treatment (Fig. 7).

### Treatment

The patient was administered systemic antibiotics and glucocorticoids for treatment.

Ceftriaxone sodium (2 g) and dexamethasone (10 mg) were administered via intravenous drip once daily, accompanied by hydroxymethazolin and mometasone furoate nasal sprays for antiinflammatory treatment for 5 days. The planned tympanic membrane incision was cancelled as the patient’s symptoms significantly improved the following day.

### Outcome

Within 1 week, the patient’s hearing levels normalized, nystagmus intensity gradually decreased, and vertigo disappeared during the course of treatment.

## Discussion

Serous labyrinthitis is diagnosed in patients with acute otitis media (AOM) who develop vertigo with spontaneous nystagmus and/or sensorineural hearing loss. The pathogenesis involves invasion of bacterial toxins (from acute/chronic otitis media, perilymphatic fistula, or meningitis), labyrinthine fluid contamination by blood, or tissue injury [6]. Sensorineural hearing loss and vertigo may arise from labyrinthine irritation induced by toxic or inflammatory mediators [7]. Recent clinical practice emphasizing early administration of optimized antibiotics for AOM has led to a notable decrease in the incidence of labyrinthitis [2].

In this clinical case, the patient presented with an upper respiratory tract infection complicated by AOM, with dizziness induced by vigorous nose blowing. It is postulated that external forces promote the transmembranous translocation of inflammatory mediators and bacterial toxins across the round and oval windows into the inner ear. Initial therapeutic management included antibiotics, glucocorticoids, and drainage procedures. Remarkably, the patient exhibited significant amelioration of ear fullness, hearing impairment, and dizziness within 24 h following treatment initiation.

Serial audiological monitoring was performed to track the patient’s hearing status. Although the patient reported satisfaction with the treatment, residual high-frequency tinnitus persisted after the procedure. She was diagnosed with persistent high-frequency sensorineural hearing loss, a condition that compromises high-frequency sound perception and potentially affects daily communication and quality of life. The middle and inner ears exhibit functional interactions, with potential pathways including hematogenic, lymphatic, oval window, round window (RW), or bony fistula routes. Substantial evidence from human temporal bone histology and animal experiments confirms that, beyond the round window membrane (RWM), no significant access pathways to the inner ear exist. The RWM serves as the sole soft tissue barrier between the middle and inner ears [8]. Serous labyrinthitis, a nonsuppurative inflammatory condition, is characterized histopathologically by endolymphatic hydrops, eosinophilic labyrinthine fluids with high protein content, and serofibrinous strands in labyrinthine spaces, all of which occur without inflammatory cell infiltration. Kaya *et al*. reported a significant reduction in the outer hair cell count within the lower basal, upper basal, and lower middle cochlear turns. In a study of 20 human temporal bones with serous labyrinthitis, no significant loss was observed in inner hair cells, spiral ganglion cells, spiral ligament fibroblasts, or the areas of the stria vascularis and spiral ligament [6, 8]. Therefore, we postulate that persistent high-frequency sensorineural hearing loss may stem from middle ear inflammation infiltrating the inner ear through the round window membrane (RWM), with preferential involvement of the cochlear basal turn. The severity of hearing loss was positively correlated with proximity to the RWM, with a shorter distance being associated with more profound hearing impairment.

This study systematically characterized progressive vestibular functional derangements in a patient with severe labyrinthitis. Serial eye movement assessments were conducted using VNG to analyze the nystagmus dynamics. Spontaneous nystagmus maintained a unidirectional orientation toward the affected side, with its intensity gradually attenuating, findings consistent with preexisting scholarly evidence [3, 9]. Irritative nystagmus may emanate from toxic or inflammatory mediators infiltrating the perilymphatic space, potentially compromising afferent nerve fibres. In addition to spontaneous irritative horizontal nystagmus, the patient demonstrated notable positional upbeat nystagmus lacking torsional components during both Dix–Hallpike and Roll maneuvers on the affected side (Fig. 3A). Posttreatment, the horizontal nystagmus gradually abated, and upbeat nystagmus resolved completely.

Upbeat nystagmus is infrequent in individuals with peripheral vestibular disorders and is generally classified as central vestibular nystagmus. This phenomenon has been documented in a range of central nervous system pathologies, including ischemic strokes, hemorrhagic lesions, neoplasms, multiple sclerosis, Wernicke encephalopathy, epileptic disorders, brainstem encephalitis, Creutzfeldt–Jakob disease, Behcet syndrome, meningitis, Chiari malformation, and cerebellar degeneration [10–13]. Mastoid CT and brain MRI were performed in all patients to exclude semicircular canal dehiscence and central vestibular pathologies.

To further elucidate the origin of positional upbeat nystagmus, we used vHIT, a novel diagnostic modality for evaluating the functional integrity of the three semicircular canals, and by extension, the superior and inferior branches of the vestibular nerve [14, 15]. The vHIT hinges on the vestibulo–ocular reflex (VOR), a neurophysiological mechanism that maintains visual fixation on a stable target during head movements. Notably, we documented a profound reduction in anterior semicircular canal VOR gain on the affected side, in the absence of compensatory saccades, with normalization following treatment. It was initially postulated that inflammation-mediated injury to superior vestibular nerve (SVN) afferent fibers underlies this observation. Preclinical and clinical studies have established that semicircular canal damage innervated by the SVN is more severe and frequent than that innervated by the inferior vestibular nerve in patients with vestibular neuritis [16, 17]. Anatomically, the SVN, which innervates the horizontal and anterior semicircular canals, utricle, and part of the saccule, has a longer average length than the inferior vestibular nerve (IVN). Anatomical studies have shown that the bony canal of the SVN is significantly longer than that of the IVN, with a length ratio of approximately sevenfold. The superior canal features a greater proportion of bone spicules and interspersed reticular bone structures. These anatomical characteristics may predispose the superior division of the vestibular nerve to entrapment and ischemia secondary to inflammatory swelling from primary infection [16, 17].

Following meticulous analysis, two areas of diagnostic ambiguity were identified. First, the patient demonstrated persistent right-beating horizontal and/or upbeat nystagmus without torsional elements, findings conflicting with the clinical hallmarks of peripheral vestibular pathology [18]. Moreover, horizontal spontaneous nystagmus was exacerbated in the supine position and during left head rolling, with a right-sided null point identified, findings congruent with a hypointense cupula [19, 20]. The hypointense cupula condition renders the cupula gravity sensitive, causing it to deflect along the axis aligned with the gravitational force. The semicircular canals (SCCs) are activated or inhibited on the basis of head position. Anatomically, the cupula is a gelatinous, leaf-like structure within the SCC ampullae of vestibular organs. Under normal conditions, the cupula has the same specific gravity as the surrounding endolymph and remains stationary unless stimulated by head movements, such as rotational motions (such as nodding “yes” or shaking “no”) or lateral tilting [21]. Endolymphatic flow deflects the cupula, eliciting excitatory or inhibitory signals in the respective canal. When the cupula has a lower specific gravity than the surrounding endolymph, gravitational force causes deflection, leading to persistent positional nystagmus—an effect driven by the buoyancy of the cupula relative to the endolymph.

It is postulated that microcirculatory compromise and infection disrupted the blood–labyrinth barrier in this patient with severe labyrinthitis, leading to plasma protein extravasation from inner ear vessels into the endolymph and increasing the specific gravity of the endolymph. Chemically derived substances from degenerated, swollen, inflammatory cells and endolymphatic otoconial particles may contribute to hypointense debris [22, 23]. Consequently, this leads to the establishment of a hypointense cupula. Understanding the spatial orientation of the semicircular canal planes within the temporal bone and the cupulae within the canals is fundamental to interpreting positional nystagmus in patients with hypointense cupula, as nystagmus genesis is governed by the alignment of the cupular axis with the gravitational vector, an explanation postulated in the buoyancy hypothesis.

As the anterior segment of the horizontal semicircular canal is inclined approximately 30° upwards from the horizontal plane, the hypointense cupula of the horizontal semicircular canal may undergo gravitational deflection, inducing spontaneous nystagmus. Notably, the intensity of geotropic nystagmus was greater during leftward head rotation than during rightward rotation, leading to the establishment of a diagnosis of right horizontal semicircular canal hypointense cupula [20]. Second, previous studies [14, 15] have reported that concurrent gain decay and compensatory catch-up saccades are hallmark features of abnormal vHIT responses. In contrast, this patient had a pronounced reduction in ipsilesional anterior semicircular canal gain without associated compensatory saccades, a discrepancy that challenges the typical diagnostic framework for superior vestibular neuritis. Concurrently, the patient displayed spontaneous horizontal nystagmus, whereas the horizontal semicircular canal VOR gain remained normal on the vHIT. As the intensity of horizontal nystagmus was less than that of upbeat nystagmus, we postulate two potential explanations. (1) Due to the anatomical continuity of the inner ear, a hypointense cupula was detected in the ipsilesional anterior semicircular canal, which exhibited more pronounced morphological alterations than did the lateral semicircular canal. This led to upbeat nystagmus on both sides during the Dix–Hallpike test and right head roll in the supine position, resulting in weaker responses to the angular acceleration stimuli. (2) The functional integrity of the corresponding vestibular afferent fibers was maintained, precluding the induction of compensatory nystagmus. Conversely, the morphological changes in the lateral semicircular canal cupula were less pronounced, resulting in mild horizontal nystagmus that did not compromise the responsiveness to horizontal angular acceleration. Therefore, the horizontal semicircular canal gain remained normal on vHIT.

## Conclusion

There are few reports regarding video head impulse tests in patients with labyrinthitis. We dynamically monitored the decrease in the gain of the upper semicircular canal on the affected side of the patient and investigated the mechanism of vertical upbeat positional nystagmus. We suggest that patients with acute otitis media exhibit spontaneous nystagmus toward the affected side, accompanied by decreased VOR gain, which necessitates caution against the development of severe labyrinthitis secondary to otitis media. If it represents atypical positional nystagmus, brain MRI is necessary to rule out intracranial complications, and additional vestibular function tests are necessary. Nystagmus, characterized by involuntary rhythmic oscillations of the eyes, is a significant indicator of the spread of inflammation. Failure to promptly treat serous labyrinthitis can lead to its transformation into suppurative labyrinthitis, causing irreversible damage to the inner ear. Therefore, patients with a history of otitis media should consider the possibility of serous labyrinthitis if they experience paroxysmal vertigo, significant hearing loss, and spontaneous nystagmus on the affected side. vHIT can effectively assist in assessing the extent of vestibular damage in patients with BPPV. Benign Paroxysmal Positional Vertigo (BPPV) is the most common peripheral vestibular disorder, causing brief rotational vertigo and nystagmus via specific head movements. It arises when inner ear otoconia dislodge into semicircular canals, disrupting fluid flow and brain sensory signals. Diagnosed via Dix-Hallpike/head roll tests, it is treated effectively with canalith repositioning maneuvers (e.g., Epley) for symptom resolution.

## Data Availability

The data supporting the findings of this study are available from the corresponding author upon reasonable request. All relevant data used in the analysis and presentation of this case report can be provided to ensure the transparency and reproducibility of the study.

## Abbreviations

AOM: Acute otitis media
VNG: Videonystagmography
HIT: Head impulse test
TM: Tympanic membrane
VOR: Vestibulo–ocular reflex
RWM: Round window membrane
SVN: Superior vestibular nerve
IVN: Inferior vestibular nerve
